# Progressive multifocal fibrosing neuropathy: description of a novel disease

**DOI:** 10.1186/s40478-022-01341-8

**Published:** 2022-03-16

**Authors:** Fabian A. Mendoza, Jennifer Bagley, Michael Gochfeld, Marinos C. Dalakas, John L. Farber, Sergio A. Jimenez

**Affiliations:** 1 Division of Rheumatology, Department of Medicine, Thomas Jefferson University, Philadelphia, PA 19107 USA; 2 Jefferson Institute of Molecular Medicine and Scleroderma Center, Thomas Jefferson University, Philadelphia, PA 19107 USA; 3 Department of Environmental and Occupational Health Sciences Institute, Robert Wood Johnson Medical School, Rutgers University, Piscataway, NJ USA; 4 Department of Neurology, Neuromuscular Division, Thomas Jefferson University, Philadelphia, PA USA; 5 Medical School, National and Kapodistrian University of Athens, Athens, Greece; 6 Department of Pathology and Cellular Biology, Thomas Jefferson University, Philadelphia, PA USA

## Abstract

Entrapment peripheral neuropathies are clinically characterized by sensory impairment and motor deficits. They are usually caused by mechanical injuries, but they are also a frequent manifestation of metabolic diseases, toxic agent exposure, or systemic fibrotic disorders. Here we describe the clinical, radiological, and histopathological features of a novel progressive fibrotic disorder characterized by progressive multifocal fibrosing neuropathy. We identified two patients who presented with severe and progressive peripheral neuropathic symptoms sequentially affecting multiple sites. These patients presented with severe and progressive multifocal, sequentially additive peripheral neuropathic symptoms. Extensive nerve conduction and radiological studies showed the sequential development of multifocal motor and sensory peripheral neuropathy in the absence of any exposure to known infectious, inflammatory, or fibrotic triggers and the lack of family history of compression neuropathies. Extensive clinical and laboratory test evaluation failed to support the diagnosis of any primary inflammatory or genetic peripheral neuropathy and there was no evidence of any systemic fibrosing disorder including Systemic Sclerosis, lacking cutaneous fibrotic changes and cardiopulmonary abnormalities. The clinical course was progressive with sequential development of motor and sensory deficits of upper and lower extremities displaying proximal predominance. Histopathological study of tissues obtained during nerve release surgeries showed severe perineural fibrosis with marked accumulation of thick collagen bundles encroaching the peripheral nerves. There was no evidence of vasculitic, inflammatory, or vascular fibroproliferative lesions. We suggest that the clinical findings described here represent a previously undescribed fibrotic disorder affecting peripheral nerves, and we propose the descriptive term "Progressive Multifocal Fibrosing Neuropathy" to refer to this novel disorder. Despite the inherent limitations of this early description, we hope this is would contribute to the identification of additional cases.

## Introduction

Peripheral neuropathies caused by nerve compression or entrapment syndromes manifest clinically by sensory impairment and motor deficits. They most commonly result from chronic mechanical injuries of nerve tracts in fibro-osseous canals or compression by other tissue structures, including tendons, ligaments, and muscles [1, 2]. Slow development of peripheral neuropathies is also a frequent manifestation of diabetes mellitus (DM) or toxic agent exposure [3–6]. On the other hand, peripheral neuropathies are an early clinical feature of systemic sclerosis (SSc) [7] and various related systemic disorders characterized by prominent tissue fibrosis, vascular alterations and immune-inflammatory changes including the toxic oil syndrome (TOS), eosinophilia-myalgia syndrome (EMS), nephrogenic systemic fibrosis (NSF), and eosinophilic fasciitis (EF) [8–14]. Peripheral neuropathies were particularly frequent and severe in patients with TOS who developed perineuritis followed by fibrosis of the perineurium and fibrosing entrapment neuropathies [9, 10]. Similarly, in advanced rapidly progressive diffuse SSc, fibrosing entrapment neuropathies are frequently present [7, 8].

Here, we describe two patients who developed a novel disorder characterized by severe and progressive peripheral neuropathy with multifocal and sequential involvement of multiple sites. Extensive neurological and histopathological studies demonstrated that this process was caused by encroachment of nerve tracts by the accumulation of large amounts of excessive fibrotic tissue in the absence of genetic or acquired neurological disorders or systemic inflammatory or fibrotic diseases. Therefore, it is suggested that this clinical entity represents a previously undescribed fibrotic disorder affecting the peripheral nerves.

## Methods

Both patients were referred to the Scleroderma Center at Thomas Jefferson University for evaluation of a systemic fibrosing disorder causing progressive neuropathy affecting multiple peripheral nerves. Appropriate laboratory tests for the assessment of SSc and SSc-like disorders, including autoantibody testing, nail-fold video-capillaroscopy, pulmonary function tests, high-resolution chest computed tomography, and echocardiograms were performed. Neurological evaluations, including extensive clinical and electrophysiological assessment, were performed by a neuromuscular neurologist (M.C.D); and radiological studies included resonance imaging (MRI), and ultrasonography. Histopathological studies of biopsies from affected nerves and surrounding tissue obtained during decompression surgeries were performed. The tissues were stained with hematoxylin–eosin (H&E), and Masson’s Trichrome stains, immunohistochemistry studies for inflammatory cell markers including CD3, CD8, CD20, and CD68 were performed. A search for peripheral myelin protein (PMP22) gene mutations to exclude hereditary neuropathy with liability to pressure palsies (HNPP) was performed in both patients (Athena Diagnostics, MA).

## Results

### Clinical features and auxiliary tests

#### Patient 1

**Fig. 1:**
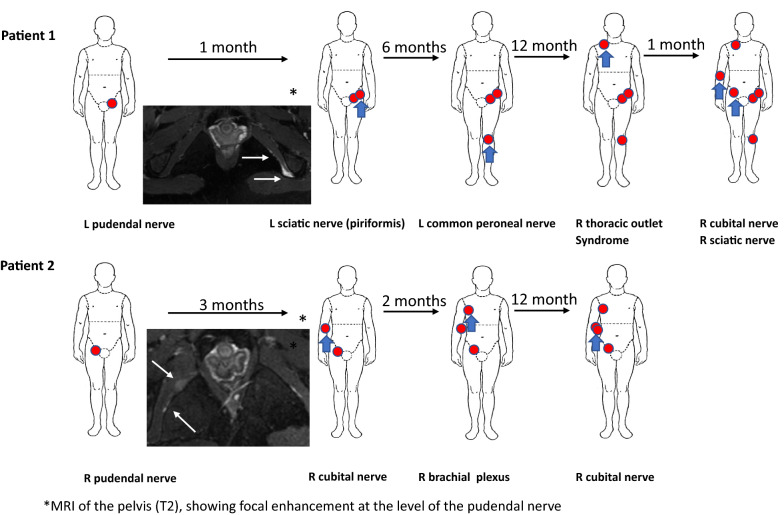
Time course and sequential development of peripheral nerve involvement. The new areas of involvement at each timepoint (red circles) are shown with the blue arrows. The inserts show MRIs (T2) of both patients displaying pudendal nerve compression by a large mass-type lesion (arrows) located near to the left ischial tuberosity in patient 1 and right iliac tuberosity and iliac bone in patient 2

**Fig. 2:**
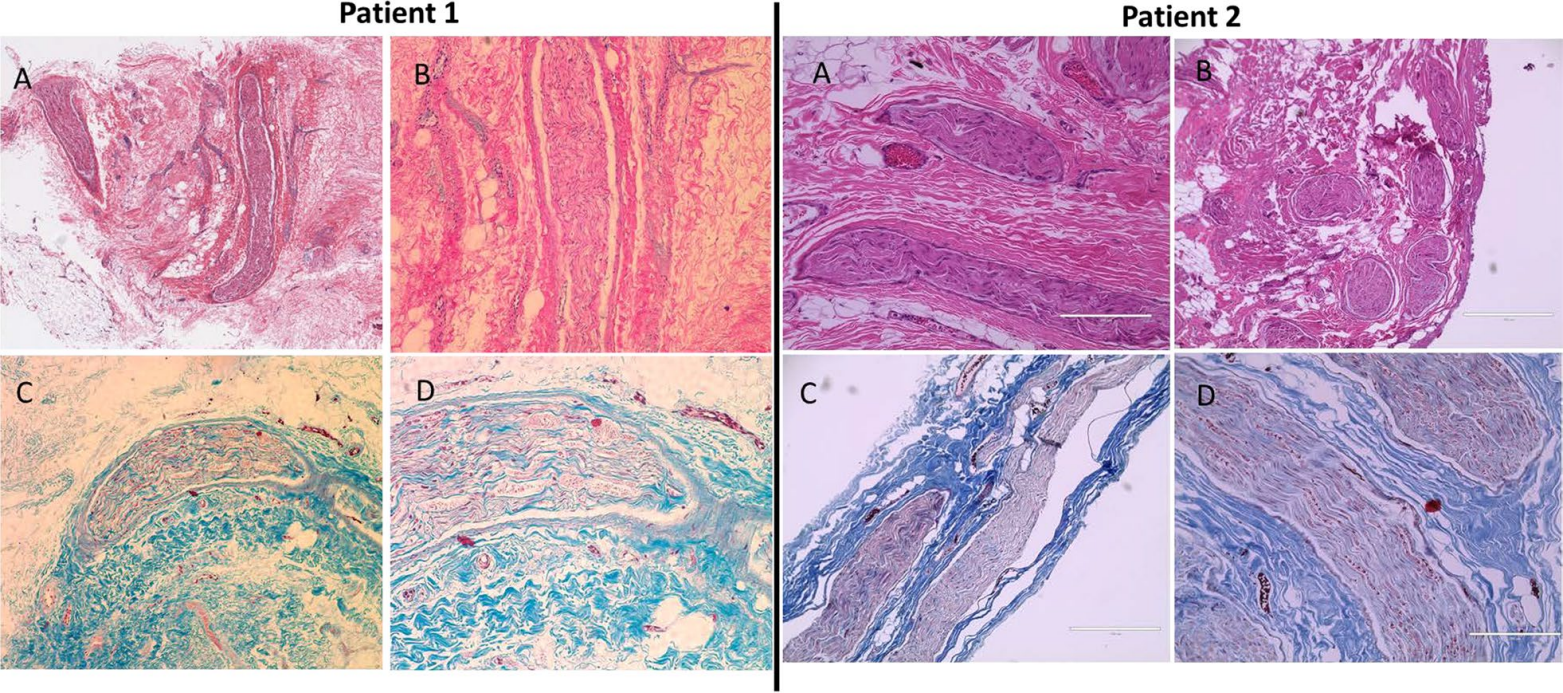
Histopathologic study of the affected tissue. Light microscopy of tissue from the left posterior femoro-cutaneous/pudendal nerve area and the right brachial area from patients 1 and 2 respectively (A and B). H&E staining (A-B) and Mason Trichrome staining (C-D) show severe fibrosis without inflammatory infiltrates encasing small nerves. Axonal swelling is also evident. There is absence of fibroproliferative, inflammatory, or vasculitic small vessel involvement. Various magnifications were used

**Fig. 3:**
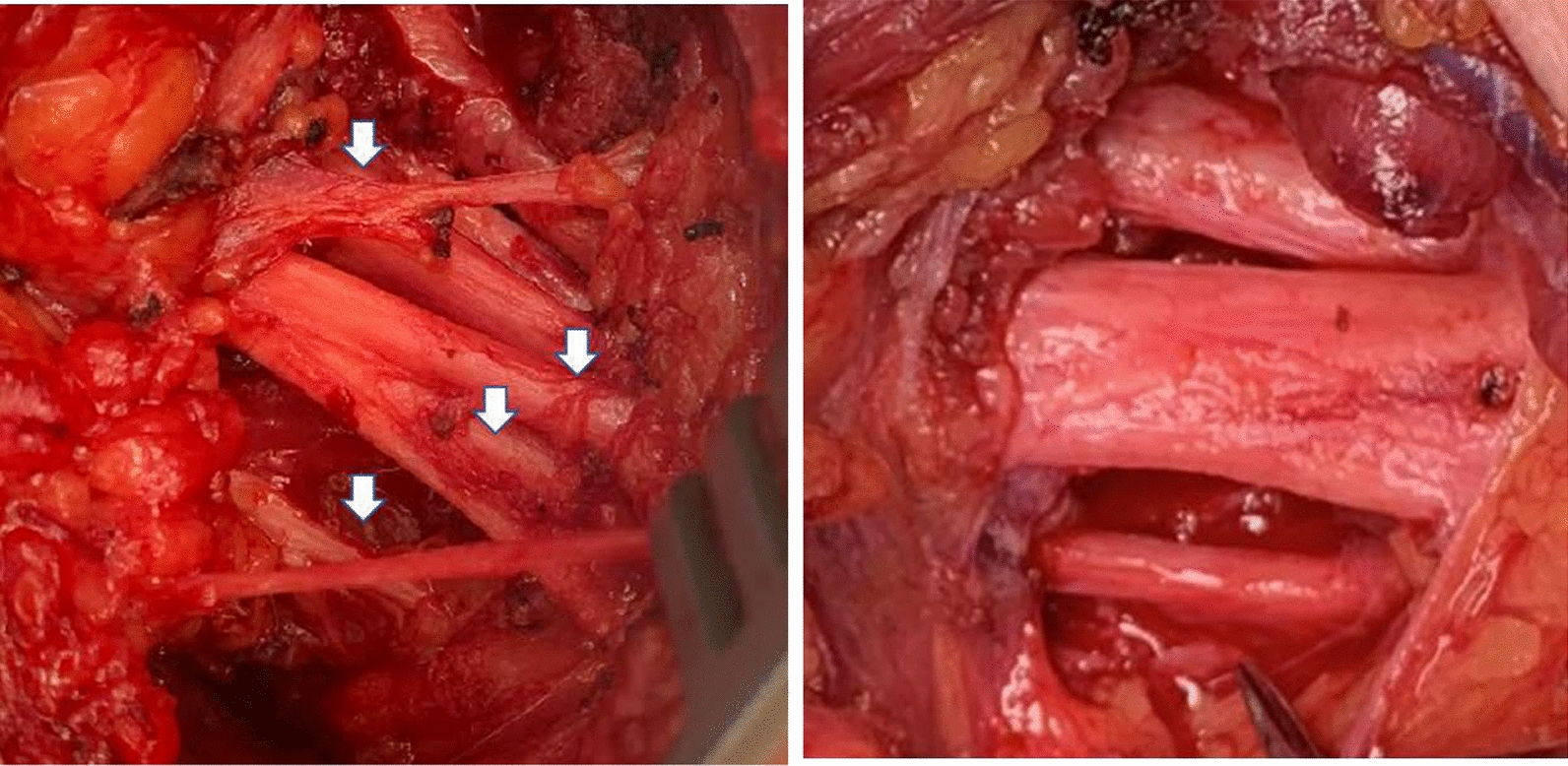
Macroscopic aspect of affected nerves. Macroscopic appearance of the right brachial plexus nerves during the surgical procedure is shown. Adhesions and perineural fibrosis surrounding and encasing nerves are displayed at the left, compared with post-surgical removal of fibrotic tissue at the right

A 37-year-old female presented with three months history of severe pain localized to the left genital area. An initial evaluation was negative for infectious etiologies. Owing to the persistence of symptoms, an MRI was performed. The study disclosed soft-tissue edema surrounding the left pudendal nerve (Fig. 1a, upper insert). She was diagnosed with left pudendal nerve neuropathy and required pudendal nerve release surgery for relief of refractory and progressive pain, achieving resolution of pain. One month later, the patient noticed left foot drop, associated with severe left buttock pain in the absence of lumbar pain and lack of trauma. Pain was aggravated on sitting, and reproduced at palpation. A positive straight leg sign was found at exam. She was diagnosed with left piriformis syndrome requiring decompression surgery. A few months later, the patient developed left common peroneal nerve compression that was treated with repeated ultrasound-guided corticosteroid injections only with partial improvement of her symptoms. One year later, a diagnosis of progressive and severe right thoracic outlet syndrome was made requiring right brachial plexus release surgery. The extensive fibrotic tissue encroachment of the brachial plexus nerve observed during the surgical procedure is shown in Fig. 3. Two months later, the patient presented with right cubital tunnel syndrome that was treated with ultrasound-guided corticosteroid injection with initial improvement. However, owing to recurrent and worsening symptoms, she required ulnar nerve release surgery. Most recently, she developed right piriformis compression syndrome, and release surgery is being considered. The sequential peripheral neurological involvement is illustrated in Fig. 1a. During all surgical release procedures, large amounts of fibrotic tissue encasing and compressing the affected nerves were found and removed. The histopathological studies of the tissues removed at surgery are shown in Fig. 2a. Electrophysiological studies performed at various times of her disease, corroborated the presence of entrapment neuropathies, and correlated with imaging and surgical findings.

#### Patient 2

A 25-year-old female patient with unremarkable medical history presented with vulvar and vaginal burning, numbness, and electric-type pain that first started three years prior to her initial visit. A comprehensive gynecological evaluation was unremarkable including negative investigation for sexually transmitted diseases. A pelvic MRI was performed shortly after the onset of her symptoms showing no structural abnormalities. She was treated with Gabapentin with partial improvement. However, few months later, her initial symptoms evolved to progressive numbness and constant neuropathic pain in the right hemi-pelvic area. A new MRI showed soft tissue “edema” and an ill-defined and enlarged right pudendal nerve (Fig. 1b lower insert). The patient failed to improve with physiotherapy and analgesics and underwent diagnostic (lidocaine) nerve blocking of the Ilioinguinal, iliohypogastric and genital branches of the genitofemoral nerve with full but transient resolution of her symptoms. Consequently, a neurectomy was performed two years following the onset of symptoms with complete resolution of pain. Three months after the procedure she developed numbness, burning, pain, and weakness of the area innervated by the right cubital nerve. She underwent electrophysiological studies (NC/EMG) that corroborated a mild right ulnar mononeuropathy. She was treated with physical/occupational therapy with stabilization of her symptoms. Two months later, the patient noted severe, spontaneous right shoulder pain radiating to the right arm. Imaging studies including ultrasound and MRI disclosed alterations consistent with right brachial plexus inflammation as well as mild edema at the right cubital tunnel. NC/EMG showed patchy involvement in brachial plexus distribution and bilateral ulnar entrapment neuropathy. There was no evidence of peripheral polyneuropathy, cervical radiculopathy, or myopathy on this study. The sequential peripheral nerve involvement is shown in Fig. 1b. Owing to rapidly progressive and worsening symptoms that included whole arm weakness, right brachial decompression surgery was performed three months later. A sample of tissue was obtained during this procedure. The histopathologic exam showed extensive perineural fibrosis encasing and compressing the visualized nerves that showed axonal swelling as shown in Fig. 2b. One year after this procedure, she developed worsening right hand and forearm motor and sensory symptoms and was treated with an ultrasound-guided nerve hydro-dissection with partial improvement.

The extensive clinical evaluation in both patients did not disclose skin thickening, digital ulcers, cardiopulmonary symptoms, mouth opening restriction, telangiectasias, or calcinosis. However, in patient 1, there was a history of mild Raynaud's phenomenon symptoms without painful episodes or digital ulcers. Detailed questioning in both patients, failed to disclose any exposure to gadolinium-based contrast agents (GdBCA), silica, organic solvents, pesticides, radiation, occupation/hobby exposures, or other identifiable environmental factors. No family history of a similar syndrome was found. Laboratory testing in both cases showed the absence of eosinophilia and tests for antinuclear antibody were negative. Repeated analysis failed to show elevation of inflammatory markers (ESR and CRP). Genetic testing did not disclose any peripheral myelin protein 22 (PMP22) mutations. An echocardiogram did not show signs of pulmonary hypertension or myocarditis, and pulmonary function tests (PFTs) were normal. There was no clinical or laboratory evidence to suggest the diagnosis of diabetes mellitus.

Given the fact that the findings of EMG/NC studies correlated with the clinical picture in both patients and provided imaging evidence of entrapment neuropathy, lumbar puncture and CSF analysis were not performed.

### Magnetic resonance and ultrasound studies

MRI of the pelvic area showed marked perineural edema surrounding the left pudendal nerve of patient 1 and right pudendal nerve in patient 2 (Fig. 1, inserts), and subsequent imaging studies of both patients including ultrasound studies of multiple nerves showed echo-density abnormalities compatible with perineural edema and fibrotic tissue encroaching multiple nerves (not shown).

### Histopathology studies

Samples of perineural tissue were obtained during the left pudendal nerve release surgery in patient 1 and during the brachial plexus surgery in patient 2. Representative images of the histopathologic results are shown in Fig. 2. H&E and Mason’s trichrome staining showed large amounts of fibrotic tissue with thick collagen bundles surrounding and encasing small nerve tracts in both patients. Axonal swelling is present. No evidence of perineural inflammatory cell infiltration was evident on the samples examined by H&E and immunohistochemistry. Extensive examination of tissue vessels failed to show any evidence of proliferative vasculopathy, vessel narrowing, or a vasculitic process.

## Discussion

There are several systemic fibrotic disorders commonly associated with peripheral neuropathy, including SSc, TOS, EF, EMS, and NSF [7–15]. However, without exception, these diseases display prominent diffuse skin and internal organ fibrotic involvement. Furthermore, these disorders display a prominent inflammatory component in affected tissues as illustrated by the inflammatory cell-induced activation of quiescent fibroblasts described in EMS [14]. In other metabolic conditions, such as DM, the progression of the entrapment neuropathies caused by remodeling of the extracellular matrix, is slow without an inflammatory reaction [16]. Although there was no clinical evidence of SSc or other scleroderma-like systemic fibrosing disease, the first patient presented Raynaud's phenomenon suggesting that vasospastic vasculopathy may be a feature of the disease. However, there was no evidence of microvascular alterations at nailfold videocapillaroscopy.

Another clinical condition mimicking some of the symptoms of the patients presented here is hereditary neuropathy with liability to pressure palsies (HNPP) [17, 18], an autosomal dominant inherited disorder usually caused by under-production of PMP-22 due to a partial deletion on chromosome 17 [17–19]. However, fibrotic lesions have not been described in this condition. Importantly the clinical phenotype of both patients was inconsistent with HNNP as their neuropathy was progressive due to a steady and continuous built-up of fibrotic tissues surrounding multiple peripheral nerves. Furthermore, the clinical manifestations were unrelated to compression events and without spontaneous improvement. Furthermore, both patients were tested for HNPP related mutations (PMP22) and the gene sequencing studies failed to show any abnormalities. In both cases there was clear evidence of both sensory and motor neurologic involvement, thereby excluding multifocal motor neuropathy that presents only with motor involvement [20].

Given the fact that several systemic fibro-inflammatory conditions have been related to an exogenous triggering agent, an extensive and detailed inquiry was performed but failed to disclose any toxic exposure. Although patients with more than one neuropathic compressive syndrome affecting the same extremity, usually associated with thoracic outlet syndrome have been described and a “double crush mechanism” was proposed for those cases [21, 22], in the patients presented here, the asymmetric distribution of the multiple involvement of deep peripheral nerves including pelvic, pudendal and thoracic nerves cannot be attributed to thoracic outlet syndrome or a “double crush mechanism”.

An extensive review of the published medical literature indicates that there was no previously described syndrome or disease with the clinical and histopathological findings described in this report and the cases and the cases studied and reported appear to be unique. Therefore, it is suggested that this may represent a novel clinical entity descriptively termed "Multifocal Progressive Fibrosing Neuropathy". The most important features distinguishing Multifocal Progressive Fibrosing Neuropathy from SSc, TOS, EF, EMS, and NSF, and other neurological diseases frequently causing neuropathy are listed in Tables 1, 2. It is anticipated that the early description of this novel disorder may allow the identification of additional patients and thereby provide further information regarding its possible etiology.

**Table 1:** Differences in the clinical features of selected systemic fibrotic diseases with Progressive Multifocal Fibrosing Neuropathy

	Nerve entrapment	Raynaud’s phenomenon	Sclerodactyly	Skin fibrosis	Lung/heart involvement	ANA/specific Autoantibodies
Progressive Multifocal Fibrosing Neuropathy	Present	Present	Absent	Absent	Absent	Absent
Systemic Sclerosis	Present	Present	Present	Present,	Present, frequently	Present
Eosinophilia Myalgia Syndrome	Present	Absent	Present	Present, diffuse	Present	Absent
Nephrogenic Systemic Fibrosis	Present	Absent	Absent	Present, varies	Present	Absent
Toxic Oil Syndrome	Present	Rare	Present	Present	Present	Absent

**Table 2:** Differences in the clinical features of selected neurological diseases with Progressive Multifocal Fibrosing Neuropathy

	Multiple Nerve involvement	Hereditary Component	Clinical features	History of trauma	Histopathology
Progressive Multifocal Fibrosing Neuropathy	Present	None	Sensory and motor loss	Absent	Marked Fibrosis in epineurium, perineurium. & endoneurium
HNPP (Hereditary neuropathy with liability to pressure palsies)	Present	Autosomal Dominant	Sensory and motor loss	Present	Multifocal thickening of peripheral myelin
Multifocal Motor neuropathy	Present	None	Motor loss only. Predominantly upper extremities	Absent	Multifocal fiber degeneration, altered size distribution, regenerating fibers
Diabetic Neuropathy	Present	Varies	Sensory and motor loss	Present (Microtrauma)	Mild fibrosis

Finally, we can hypothesize that owing the absence of inflammation in immunopathological studies but significant fibrosis, these patients could benefit from the use of antifibrotic drugs.
